# Gender and social geography: Impact on Lady Health Workers Mobility in Pakistan

**DOI:** 10.1186/1472-6963-12-360

**Published:** 2012-10-16

**Authors:** Zubia Mumtaz

**Affiliations:** 1 Assistant Professor, School of Public Health, University of Alberta, 3–309 Edmonton Clinic Health Academy, 11405 – 87 Ave, Edmonton, AB T6G 1C9, Canada

**Keywords:** Reproductive health services, Primary health care services, Pakistan, Family planning, Community health workers, Lady health worker, Gender, Women’s mobility, Social geography, Mixed methods

## Abstract

**Background:**

In Pakistan, where gendered norms restrict women's mobility, female community health workers (CHWs) provide doorstep primary health services to home-bound women. The program has not achieved optimal functioning. One reason, I argue, may be that the CHWs are unable to make home visits because they have to operate within the same gender system that necessitated their appointment in the first place. Ethnographic research shows that women’s mobility in Pakistan is determined not so much by physical geography as by social geography (the analysis of social phenomena in space). Irrespective of physical location, the presence of *biradari*^a^ members (extended family) creates a socially acceptable ‘inside space’ to which women are limited. The presence of a *non-biradari* person, especially a man, transforms any space into an ‘outside space’, forbidden space. This study aims to understand how these cultural norms affect CHWs’ home-visit rates and the quality of services delivered.

**Design:**

Data will be collected in district Attock, Punjab. Twenty randomly selected CHWs will first be interviewed to explore their experiences of delivering doorstep services in the context of gendered norms that promote women's seclusion. Each CHW will be requested to draw a map of her catchment area using social mapping techniques. These maps will be used to survey women of reproductive age to assess variations in the CHW's home visitation rates and quality of family planning services provided. A sample size of 760 households (38 per CHW) is estimated to have the power to detect, with 95% confidence, households the CHWs do not visit. To explore the role of the larger community in shaping the CHWs mobility experiences, 25 community members will be interviewed and five CHWs observed as they conduct their home visits. The survey data will be merged with the maps to demonstrate if any disjunctures exist between CHWs’ social geography and physical geography. Furthermore, the impacts these geographies have on home visitation rates and quality of services delivered will be explored.

**Discussion:**

The study will provide generic and theoretical insights into how the CHW program policies and operations can improve working conditions to facilitate the work of female staff in order to ultimately provide high-quality services.

## Background

Millennium Development Goals 4 and 5 aim to reduce child and maternal mortality rates by two thirds by 2015. Progress to date suggests countries with the highest burden will fail to meet these targets [1]. Although a number of factors are responsible for this failure, one key reason is a lack of health care providers necessary to deliver the simple and cost-effective strategies that can dramatically reduce maternal and child mortality [2,3]. The density of health workers (doctors, nurses, midwives) is inversely associated with maternal, infant and under-five mortality [4]. Most developing countries are, however, currently experiencing acute human resource shortages in the health sector [2]. The high cost of training physicians and nurses and the low use of services based in health facilities, together with the recent renewed interest in primary health care, has rekindled interest in Community Health Workers (CHW) [5,6].

CHWs were envisaged as a cornerstone of the primary health care approach as far back as 1978 in Alma Ata [7]. Enthusiasm for such workers diminished in the 1990s as evidence of their ineffectiveness mounted [8,9]. Although most programs were gradually phased out in Africa and Latin America [10], they remained a common policy and programmatic response in South Asia, mainly because women’s seclusion, widespread in this context, is a key barrier to their travel to health and family planning facilities [11]. One such program is the National Program for Family Planning and Primary Health Care in Pakistan, also known as the Lady Health Worker (LHW) program. It consists of 100,000 locally resident *female* CHWs, who provide family planning, antenatal and child health care. Program coverage is about 50-60% of rural areas and urban slum populations [12].

Despite its success, the functioning of the LHW program remains suboptimal, as indicated by the following statistics. Pakistan is the sixth most populous country in the world, projected to become the fourth by the year 2040 [13]. Survey evidence suggests there is high latent demand for family planning services in Pakistan: 52% of women aged 15–49 want to stop childbearing and another 20% desire to delay their next birth for two or more years [14]. The current low rate of contraceptive use (29%) suggests that it is the family planning services that are inadequate and failing to meet demand. The LHWs, a key pillar of the family planning program in Pakistan, is cited by only 8% of users as their source of contraceptives [11].

Program evaluators have largely focused on program inputs and processes to explain why program coverage remains suboptimal [15]. I hypothesize one additional reason: the LHWs are unable to make home visits because they are, like the women they seek to serve, subject to the same gendered restrictions that limit women’s mobility in this context. Previous ethnographic research in Pakistan suggests women’s mobility is a complex behaviour, determined not so much by physical geography (*i.e*., location characteristics) as by social geography (*i.e*., the analysis of social phenomena in space) [16]. Irrespective of physical location, it is the presence of *biradari*^a^ members in a space that creates a socially acceptable ‘inside space’ to which women are limited. The presence of a non*-biradari* person, man or woman, transforms any space into an ‘outside space’ and is therefore forbidden. Women can visit a relative’s house that involves a lonely 45-minute walk in the fields, but cannot visit a non-*biradari* house, a five-minute walk away. Gender norms also dictate women should have minimal extra*-biradari* social interactions [13].

To date, little attention has been given to how these complex gendered norms and values regulating women’s mobility, visibility and social interactions affect a *female* CHW’s ability to deliver family planning services in people’s homes. These workers are ultimately required to operate within the same gender system that necessitated their appointment in the first place. How do gender values of women’s seclusion, in all their complexity, affect a CHW’s ability to travel on the village lanes and roads? How do gendered rules of social interaction beyond the *biradari* affect a CHW’s ability to visit non-*biradari* households comfortably? Would an assignment of households based on a CHW’s social geography improve home visitation rates, quality of services and promote better health outcomes for the people? These questions become crucial in the context of family planning services given that fertility is an emotive, deeply gendered and power-related issue [17].

The aim of this study is to conceptualize and develop a framework for understanding and addressing barriers faced by *female* CHWs as they struggle to provide doorstep family planning services in rural Pakistan, a milieu characterized by gendered norms, values and structures that restrict women’s mobility and social interactions. The study will generate potentially transformative new understandings of gendered processes that disadvantage female CHWs’ mobility and provision of family planning services at the doorstep. It will also provide generic and theoretical insights into how CHW program policies and operations can improve working conditions to better facilitate the work of female staff so as to ultimately provide high-quality family planning services. It is important to pursue this research now, as the recent renewed interest in primary health care [6], together with global shortages in human resources, has created a positive policy climate open to new ideas around the potential contribution of LHWs to the delivery of primary health care services.

### Research objectives

The objectives of this project are to:

1. Gain a comprehensive understanding of the ways in which gendered norms that govern women’s mobility, visibility and cross-*biradari* social interactions in the district of Attock, Punjab may constrain LHWs’ ability to make home visits and interact with non-*biradari* women and men and how these issues affect program operations and outputs.

2. Explore and document if and how the LHWs maneuver around these gendered barriers to their mobility and social interactions with non-*biradari* women and men. In particular, there is a need to 'unpack' the ways in which LHWs overcome these barriers as this may provide the key to addressing program delivery issues.

3. Contribute to the design of potential policies and strategies to address and overcome the gendered barriers LHWs face to facilitate their work, increase their effectiveness and improve service delivery.

## Methods/design

Using a mixed methods approach, data will be collected in three overlapping phases in district Attock, Punjab, Pakistan. Twenty LHWs will be randomly selected from the district LHW program database.

1. **Phase 1**: Using a focused ethnographic approach, I will conduct 45 in-depth interviews [18] and six focus group discussions [19] with randomly selected LHWs (20 interviews) from the program database (available from the District Health Office) and community members, both women and men (25 interviews and six focus groups).

2. In **Phase 2:** the 20 randomly selected LHWs will draw maps of their catchment areas using social mapping methods [20]. Social mapping methods produce an emic perspective of their social geography and its boundaries, as well as other socio-economic and power structures that outside researchers cannot observe directly. Researchers will complement these methods with observations of five LHWs during their daily rounds. The houses she visits, her demeanor as she walks down village lanes, the behaviour of other people towards her, and how she is generally treated will be recorded.

3. In **Phase 3:** a survey will be conducted. The universe for the survey will be the assigned households of the 20 LHWs who participated in Phase 2. The maps they will draw will be used to demarcate their catchment areas. It is estimated that using a sample size of 760 households (38 per CHW) will have the power to detect, with 95% confidence, households LHWs do not visit.

### Data analysis

1. Survey data will be analyzed using Stata 12 [21] to assess if rates of contraceptive use, LHW home visitation rates, and provision and quality of family planning services provided vary by *biradari* relationship between the LHW and the respondent. Multiple regression methods will be used to assess if there are significant differences in household visitation rates, quality of services provided, and in outcomes (such as contraceptive use rates, controlled for potential confounders) by *biradari* relationship between the respondents and LHWs.

2. The survey data will then be merged with the paper schematic maps obtained in social mapping exercises to query for any relationships between the LHWs’ social and physical geographies, home visitation rates, and quality of services provided. Firstly, the maps will be digitized in a Geographic Information Systems package (ArcView) [22]. They will then be merged with survey data to visually assess any disjunctures between LHWs’ social geography and the physical geography of their assigned household. Are visitation rates to households that fall outside the boundary of a LHW’s social geography different from those that fall within the boundary?

3. Qualitative data: The objective of the qualitative data is to explore (1) how the LHWs, as women, experience the gender values around women’s mobility in their delivery of doorstep services; (2) what factors predict positive and negative experiences; and (3) the role of the larger community in shaping a LHW’s experience. A database of the transcribed interviews and focus groups will be created. Using a social constructivist, interpretative approach [23], data will be coded and broad themes identified. Initial coding will be guided by the stated research objectives and later by additional concepts as they emerge. Narratives from different sources will be merged to describe typical experiences and behaviours, although the atypical will also be accounted for and alternate explanations of the phenomena will be carefully considered [24].

Data analysis will be an on-going and iterative process throughout all phases of the data collection process, as early identification of themes will allow a fuller probing of unanticipated concepts and variables in upcoming interviews [25]. Interpretive accuracy will be assessed using triangulation of findings, peer debriefing with colleagues and respondent validation [26].

### Ethics

Ethics approval has been obtained from the University of Alberta Health Research Ethics Board and the Pakistan Medical Research Council National Bioethics Board. Confidentiality, voluntary, informed participation and safety of participants will be given priority during the research process. A potential discomfort for the participants associated with this research is that the LHW may perceive the research as an evaluation of her performance. To reduce LHW apprehensions, we will inform them in detail the purpose of the study and assure her that her identity will never be revealed. Ethics clearance has been obtained on the basis of oral consent, will be obtained from both the LHWs and community members, and the researcher will fill in and sign the forms to confirm that oral consent has been obtained. It is important to seek oral consent only as the research will be conducted in a rural area with low levels of education. Requests for signatures in this context are viewed with a high degree of suspicion for it indicates a legal document is being signed, over which they perceive they will have little control. Even the CHW, although relatively better educated, are expected to be hostile to the idea of signing consent forms.

## Discussion

The concept of minimally trained CHWs as health care providers is a subject under intense debate. The failure of such programs in Africa and Latin America in the 1980s has led many to oppose the idea [8]. Other researchers argue that there is, today, a paucity of good quality research documenting the effectiveness of CHWs and the reasons they failed [6].

The findings of the present research will provide crucial insights into some key socio-cultural reasons that may be limiting program effectiveness. If translated into effective program policy and action, the findings have the potential to transform the program into a role model of success. Such success will provide much needed evidence to support a key component of the Alma-Ata primary health care approach, that health workers with short periods of training can bring health care as close as possible to where people live. It will also provide evidence to support community health programs currently being developed in Ethiopia, Kenya, India, Uganda and South Africa.

## Endnote

^a^A *biradari,* at its simplest, is defined as a group of households related by blood. It is the basic social, class, economic and political unit in Pakistani society.

## Abbreviations

LHW: Lady Health Worker; CHW: Community Health Worker.

## Competing interests

The author has no conflicts of interest to declare.

## Authors’ contributions

ZM conceptualized the study and prepared the manuscript.

## Authors’ information

**ZM (**MBBS, MPH, PhD)**,** Alberta Heritage Foundation for Medical Research Population Health Investigator and Assistant Professor, School of Public Health, University of Alberta. She specializes in gender and reproductive health issues with a particular focus on women’s access to reproductive health services and inequities in reproductive health policy, design and delivery of services.

## Funding

This study is funded by the Killam Foundation, Canada.

## Pre-publication history

The pre-publication history for this paper can be accessed here:

http://www.biomedcentral.com/1472-6963/12/360/prepub
